# Monomaniacs

**Published:** 1876-10

**Authors:** 


### *MONOMANIACS.*

We once read of a wealthy young man who was so careful of his health that he did nothing else but look after it. His physician was constantly with him. A careful and experienced nurse was always in attendance, as though he were an invalid. He caused himself to be weighed before and after each meal, in order that he might regulate the quantity of food to the requirements of his system. Any considerable loss of weight in a single month filled him with alarm. Of course the man was a lunatic, and his physician a dunce, not to have discovered it the first moment he saw him. This was a case of extreme caution; of monomania; but it was in the right direction. The opposite extreme, of recklessness of health or safety, would have brought his life to an end in a week.

We have no patience with the man, who, on a warm, muggy day, insists on closing the only open window in the car, particularly when every seat and all the standing room is occupied, leaving no chance for retreat. We might quietly enjoy a tilt that would cause our neighbor's head to punch a hole through the glass, were he not a monomaniac. Here is a man who imagined he was taking the very best care of his health when he shut out the pure fresh air, and for an hour re-breathed the air poisoned by exalations from the lungs, bodies and clothing of sixty or seventy persons. There are many such people. They dread a current of pure fresh air, preferring to breathe carbonic acid gas. Were one of them to eat a piece of "roast beef rare"—he would imagine he had "tape-worms" in less than twenty-four hours after; but he daily and cheerfully submits to having the inner surface of his stomach and intestines rasped by "graham" bread and like abominations. These are only a few of the many peculiarities of such persons.

They are confirmed monomaniacs, and beyond the reach of reason or of the most conclusive evidence. They have faith in nothing but themselves. Nothing so surely makes a person selfish, suspicious, misanthropic and irreverent, as a protracted course of low diet.

Our lamented friend—Dr. Hall—used to laughingly refer to these people as his "tutors." He said that such a one would cautiously enter the office as if he feared to break down the door. After looking at the doors and windows long enough to satisfy himself that no air could enter the room, he would preface his utterances with a few feeble coughs, and then state his case. He would snappishly dissent from any opinion the Doctor had a chance to edge in, and wind up the interview, or rather call, by a fierce tirade against doctors, and "the rest of mankind" generally. Verily; "a sound mind in a sound body," is a treasure "more precious than the golden wedge of Ophir."

